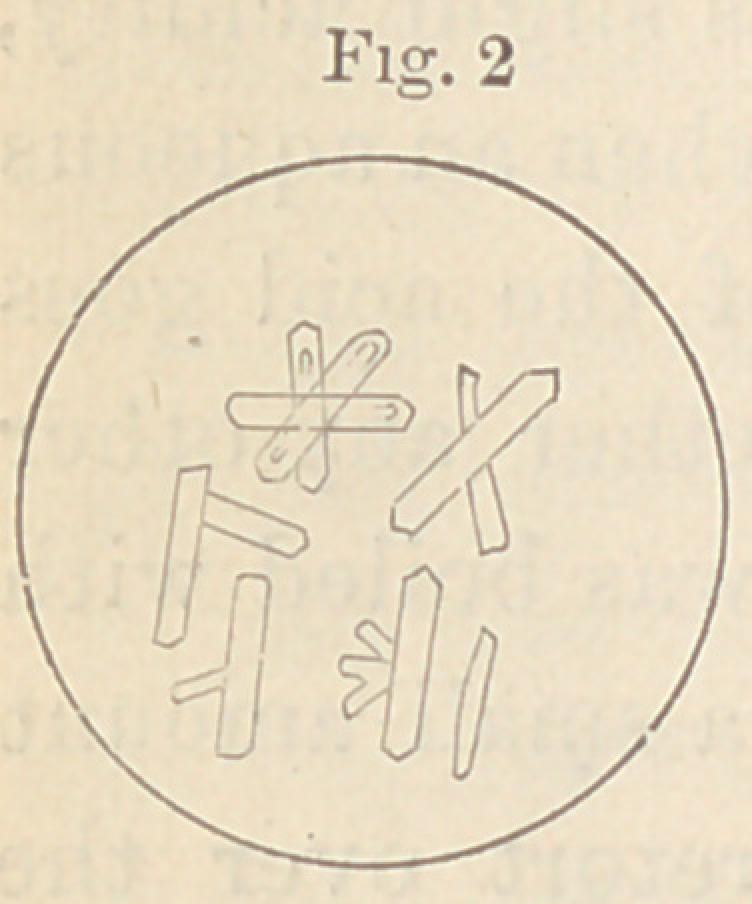# Fermentation in the Human Mouth; Its Relations to Caries of the Teeth

**Published:** 1884-03

**Authors:** W. D. Miller

**Affiliations:** Berlin, Germany


### Independent Practitioner.

VOL. V. MARCH, 1884. No. 3.

### Original Communications.

### FERMENTATION IN THE HUMAN MOUTH; ITS RELATION TO CARIES OF THE TEETH.

BY DR. W. D. MILLER, BERLIN, GERMANY.

(Continued from page 65.)

In addition to the experiments described in the preceding number of this journal, I add the following: A sound bicuspid tooth was sawed into sections, varying from one-third to one m. m. in thickness, and an equal number of these sections placed in each of two test-tubes. Into one of these test-tubes were then brought five c. c. of a perfectly neutralized two per cent. aqueous solution of beef extract; into the other the same solution, with the addition of 0,2 cane sugar. Both tubes with their contents were then sterilized, and, upon cooling, infected from a pure culture of the fungus under consideration.

The solution in the second tube became acid in a few hours; not so, however, with that in the first tube, it being non-fermentable. At the end of one week the thinner sections in the second tube were so far softened that one of them, removed for examination, could be easily bent between the fingers. At the end of the second week all but the thicker sections were completely decalcified. One of these sections was now placed upon the freezing microtome and made into cuts, which were stained in fuchsine and mounted in Canada balsam. A microscopic examination showed that the fungi had penetrated many of the tubules to a considerable depth, the invaded tubules being at the same time slightly extended. At the close of the third week the invasion was found to have become much more extensive, the tubules much dilated, and in some places the walls were broken through, leading to the formation of oval spaces or caverns in the dentine.

In short, we had a typical case of caries.

It is hardly necessary to state that the thinnest sections in the first tube, where the development of the fungus was not accompanied by an acid fermentation, did not show even the traces of softening, to say nothing of caries.

I had then produced caries by inoculating sound dentine from a pure culture of a fungus found in carious dentine, in the presence of the same fermentable substances that occur in the mouth. It seems that a clearer solution of the problem can at present scarcely be expected. Of course the thought at once suggests itself to every one that this decay is quite independent of putrefaction; all evidence points to the conclusion that putrefaction at most does nothing more than dispose of the already devitalized and much riddled remains of tissue, and we are in danger of overrating its influence, even at this stage.

Pieces of dentine in a solution kept constantly pure and sour by fermentation, not only become softened and show the microscopic changes characteristic of carious dentine, but finally, after some months, disappear altogether, as has repeatedly been the case in my cultures. From this we must infer that the process commonly known as putrefaction is absolutely essential at no stage of caries; especially is this the case in caries of enamel.

It has been intimated that the active agent in this process is nearly related to, if not identical with, the fungus of sour milk: *Bacterium acidi lactici.* The analysis of the product of fermentation will show the truth or falsity of this supposition.

The method of carrying out such an analysis will now be given. Two hundred c. c. fresh saliva are mixed with 2,0 starch and allowed to stand forty-eight hours at blood temperature; the mixture is then filtered and heated to one hundred degrees Centigrade, to stop the fermentation. This process is repeated until about a litre of the solution has accumulated. It is then placed in a retort and reduced to a volume of about seventy-five c. c. It will be very strongly acid. A few drops of this liquid are added to a thin solution of methyl-violet, and leave it unchanged; from this we conclude that we have to deal with an organic acid, as an inorganic acid would turn it first blue, and then green. Since the acid did not distill during the prolonged boiling, we may set it down as non-volatile; hence a non-volatile, organic acid. The distillate was very slightly acid; we will call it distillate number one, as we wish to refer to it again.

The solution was further reduced in volume to about forty c. c. over the water-bath, and then transferred to a large glass vessel, briskly shaken with one and one-half to two litres of sulphuric ether, and allowed to stand until the ether became perfectly transparent. This was then filtered into a large retort and distilled, proper precautions being observed to prevent accidents. When the volume had been reduced to about fifty c. c. the solution was filtered into a porcelain vessel, and still further reduced over the water-bath. A portion of the solution tested in the short tube of a Mitscherlich double-shadow polaristrobometer gave, as a mean of nine readings, a rotation of the plane of polarization equal to 0,015 degrees, or 0° 0′,9. In other words, the solution was optically inactive, the 0° 0′,9 being far within the range of the error of experiment, especially as the solution was not absolutely transparent.

An excess of freshly prepared oxide of zinc was then added to the solution, and the whole slowly and carefully boiled, water being added as it was found necessary, till the reaction became neutral, or nearly so, filtered into a large glass evaporating dish, and put away at the temperature of the room for the salt to crystallize. A drop of this solution placed upon a glass slide gave, upon crystallization, the forms seen in figure one, which are at once recognized as crystals of lactate of zinc. In a few days a quantity of a whitish crystalline powder had formed. This was placed upon a filter, the mother-liquid squeezed out, washed in absolute alcohol, dissolved in hot water, re-crystallized and dried over sulphuric acid; it then weighed 0,343. After exposing to a temperature of one hundred degrees Centigrade, or a little more, till the weight became constant, it weighed 0,2816; it lost accordingly 17,9 per cent.* of water of crystallization, corresponding to three molecules of water. The salt was then dissolved in water, the zinc precipitated as carbonate and burned. The burned mass (zinc oxide) weighed 0,0970. We have consequently:

Fig. 1

Substance analyzed (a zinc salt) = 0,343
Oxide of zinc = 0,097

The zinc oxide is seen to be equivalent to 28,2 per cent. of the substance analyzed.

The formula for the inactive ethylidene lactate of zinc is—

$$\left.\begin{matrix} C_3\,H_5\,O_3 \\ C_3\,H_5\,O_3 \end{matrix}\right\} Zn + 3\,H_2\,O, = 243 + 54.$$

Dried at ordinary temperature it contains 27,3 per cent. zinc oxide. The result obtained from the analysis differs, therefore, from that deduced from the formula by less than one per cent., and settles beyond doubt the fact that the substance analyzed was the lactate of zinc, or that the acid generated by the fermentation is lactic acid, or, more exactly, inactive ethylidene lactic acid, since, as shown above, the acid solution was optically inactive, and the zinc salt contained three molecules of water of crystallization. The salt was furthermore soluble in sixty-two parts water at fourteen degrees Centigrade.

I repeated the analysis with the following solution:

* Theoretically 18,2, or 0,3 per cent. more.

Water, 1000 c. c.
Saliva, 300 c. c.
Bouillon, 200 c. c., made by boiling 125,0 beef ten minutes in 300 c. c. of water.
Sugar, 10,0.

This solution being slightly acid was neutralized with the carbonates of lime and sodium, sterilized, and infected from a pure culture of the fungus in question.

It was treated throughout exactly in the manner above described, except that the zinc salt was converted into the sulphide instead of the carbonate, and burned with powdered sulphur in a stream of hydrogen. The result was as follows:

| | | |
|---|---|---|
| Substance analyzed, | = | 1,0540 |
| Zinc sulphide, | = | 0,415 |
| Zinc, | = | 26,38 per cent. |
| instead of | | 26,74 per cent., as deduced from the |

formula, a difference of only one-third of one per cent.

In this case the substance was dried at one hundred degrees Centigrade before weighing, and the formula becomes

$$\left.\begin{matrix} C_3\,H_5\,O_3 \\ C_3\,H_5\,O_3 \end{matrix}\right\} Zn = 243.$$

One more analysis was made, using—

Water, 1000 c. c.
Liquid beef extract, 20 c. c.
Sugar, 10,0.

The result was the same, and need not be given; the two analyses above described being abundantly sufficient to show that the acid generated by the fungus in question is the common ferment, lactic acid.

Distillate number one, referred to above, owed its slight acidity, we now know, in part at least to lactic acid, since, when an aqueous solution of lactic acid is boiled, a small fraction of the acid goes over with the water. To ascertain, however, whether any other acid, especially volatile, was present, the distillate was boiled with carbonate of lime, filtered, evaporated to dryness, a small amount of dilute sulphuric acid added, and heated in a retort over the water-bath; a few drops of an oily acid came over, which, when taken upon the fingers, smelled like butyric acid; the amount, however, was so small, that no attempt could be made to analyze it.

I have been able with some degree of certainty to establish the presence of lactic acid in carious dentine, by a method theoretically so simple that it seems strange it has never been made use of before, but which, however, in practice is only carried out with great difficulty. My first and second attempts were only partially successful; the third succeeded sufficiently well to justify its description here.

In this experiment I made use of fifteen teeth, all containing considerable quantities of carious dentine, and all extracted on the day of use. The remains of food were first removed from the cavities, but none of the softened dentine; then all the softened dentine was taken out and placed in a porcelain vessel, cut or picked into fine pieces, placed in a test-tube with one c. c. of water, and two drops of a ten per cent. solution of hydrochloric acid added. Any free lactic acid in the carious dentine would remain free, and any existing in combination with lime would be set free by the hydrochloric acid. It was then gently shaken with about twenty-five c. c. sulphuric ether, and the latter, holding the lactic acid in solution was, after some minutes, poured off into a second test-tube; here it must be allowed to stand from twenty-four to forty-eight hours, till it becomes perfectly clear. It was then filtered into a porcelain dish, evaporated, a few drops of distilled water and a small quantity of *freshly prepared* zinc oxide added, gently boiled (water being added as necessary) for ten minutes, the three or four drops of liquid remaining filtered on to a glass slide, and allowed to crystallize. I obtained the forms seen in figure two.

Fig. 2

Their close resemblance to the crystals of the lactate of zinc (Fig. 1) will be seen at once. There can, in fact, scarcely be a doubt that they are lactate of zinc crystals. The lactic acid concerned in their formation must of course have existed in the carious dentine.

I have noticed in the dental journals a tendency on the part of some writers on this subject, to derive a large amount of satisfaction from the statement that, after all, what I have done to clear up the subject of dental caries was done and known long ago.

One writer even states that he might almost have said two years ago, something that I said but a few months since. Let me say, once for all, that I have too little spare time to devote any of it to the discussion of the question who *said* this or that first, or even who *might almost* have *said* something two years ago. There is perhaps no human disease about which more has been *said* than about caries of the teeth, and when the subject shall have received its final settlement there will be hundreds who may say "I told you so." Malassez and Vignal very justly say of Baumgarten, who claims priority over Koch in the discovery of the tubercle bacillus: "*Il ne suffit pas de* trouver, *il faut* prouver"—and I do not hesitate to say with reference to some of the discussions which for years have been carried on concerning the cause of dental caries: *Il ne suffit pas de deviner, il faut trouver* et *prouver.*

It is not enough to guess the cause, or guess at it; we must *find* the cause, and, having found it, *prove* that it is the cause sought for.

(TO BE CONTINUED.)

## Figures and Tables

**Fig. 1 f1:**
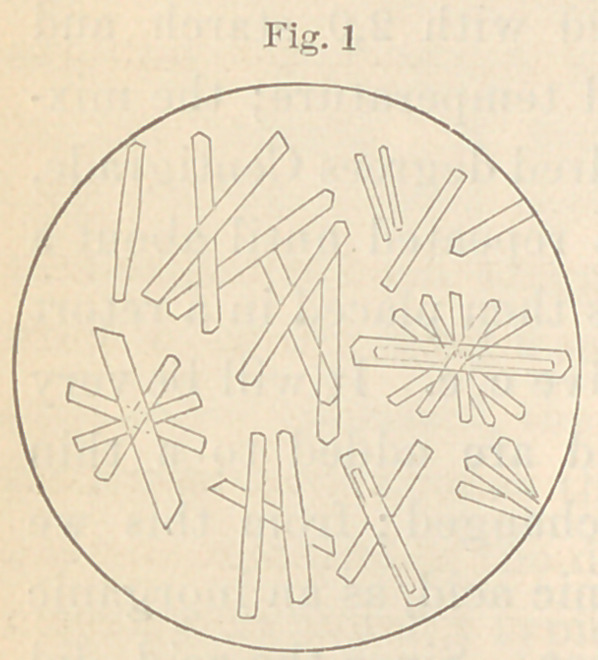


**Fig. 2 f2:**